# Essentials of Materia Medica, Therapeutics and Prescription Writing, Arranged in the Form of Questions and Answers—Prepared Especially for Students of Medicine

**Published:** 1890-02

**Authors:** 


Essentials of Materia Medica, Therapeutics and Prescription Writing, Arranged in the Form of Questions and Answers—Prepared Especially for Students of Medicine. By Henry Morris, M. D., late Demonstrator of Anatomy, Jefferson Medical College; Co-editor Beddle's Materia Medica; Visiting Physician to St. Joseph's Hospital; Fellow College of Physicians, Philadelphia, etc. Philadelphia, W. B. Saunders; London, Henry Renshaw; Melbourne, George Robertson & Co. 1889.

This is a work of 250 pages, presenting, in the form of questions and answers, a condensed outline of materia medica. It is brought down to most recent advances in this very essential department of medical science. The matter of the work corresponds to such standard text-books as Wood, Bartholow, Ruger, Brunton and others, and must prove exceedingly useful and convenient to the student of medicine.

